# Mechanical Characteristics of Soft Clay Solidified by Incorporating Granulated Blast Furnace Slag, Magnesium Oxide, and Building Gypsum

**DOI:** 10.3390/ma18081757

**Published:** 2025-04-11

**Authors:** Henggang Ji, Xiang Fan, Fan Ding

**Affiliations:** School of Highway, Chang’an University, Xi’an 710064, China; jihenggang@chd.edu.cn (H.J.);

**Keywords:** clay solidification, granulated blast furnace slag, alkaline and sulfate activator, freeze–thaw cycle, sodium sulfate solution attack, mechanical characteristic, microstructure

## Abstract

Super sulfate cement (SSC) serves as a sustainable alternative to ordinary Portland cement, offering lower carbon emissions and superior performance. Magnesium oxide (MgO) and building gypsum (BG) were utilized as activators for granulated blast furnace slag (GBFS), and together they formed SSC, which was employed to stabilize the waste soft clay (SC). The mechanical strength development characteristics of solidified clay and the types of its hydration products were investigated through mechanical experiments, including unconfined compressive strength (UCS) tests as well as microscopic experiments, such as X-ray diffraction tests and scanning electron microscopy tests. The mass ratios of GBFS, MgO, and BG were 8:2:0 (A2) and 6:2:2 (B1), respectively; these ratios were employed to stabilize the clay, resulting in solidified clay samples designated as S-A2 and S-B1. The UCS of S-B1 increased by 36.5% to 49.3% compared to S-A2 at the curing time from 7 to 91 days. The strength residual coefficients were 34.5% and 39.1% for S-A2 and S-B1, respectively, after ten wet–dry cycles. After soaking in sodium sulfate solution, the UCS of S-A2 and S-B1 decreased by 49.1% and 29.8%, respectively, compared to the unsoaked condition. The results of microscopic tests showed that the hydration products of S-B1 mainly included needle-like calcium silicate hydrate (C-S-H) gel, flaky hydrothermal gel, and ettringite (AFt) crystals. BG promoted the formation of AFt, while MgO facilitated the generation of C-S-H gel. In this study, SSC was used to stabilize the waste clay, which provided a way for the application of waste SC and SSC.

## 1. Introduction

As various types of infrastructure continue to expand in China, excavation operations for roads, bridges, and harbor projects located in coastal areas and along rivers generate a large amount of construction soft clay every year [1,2]. The soft clay in these areas exhibits characteristics of high moisture content, high compressibility, and low strength, which complicate its direct application in engineering projects [3,4]. Currently, solidification methods are considered an effective way to solve a large number of clay treatment problems [5,6]. Solidification technology refers to the mixing of waste clay with binder materials, which react with the pore water in the clay through a hydration reaction. This process reduces the clay’s high moisture content and enhances its strength, facilitating its reuse in engineering applications.

Traditionally, one approach to reusing soft clay involves mixing it with cement, which acts as a binder for the clay [7,8,9]. In light of the urgent need to reduce greenhouse gas emissions in the traditional cement industry, efforts are underway to identify environmentally friendly, low-carbon binding materials as alternatives to ordinary Portland cement (OPC) [10]. Super sulfate cement (SSC) has emerged as a prominent alternative due to its streamlined production process and decreased consumption of natural materials and energy [11,12,13]. The composition of SSC primarily includes ground granulated blast furnace slag, sulfate activators, and alkaline activators [14]. The synergistic effect of sulfate and alkaline activators can effectively activate granulated blast furnace slag (GBFS), forming hydration products such as calcium silicate hydrate (C-S-H) gel, ettringite (AFt), and gypsum [15,16].

Singh et al. [17] reported that the application of phosphogypsum as a sulfate activator for SSC yielded a cementitious material with greater strength compared to that produced using anhydrite as the sulfate activator. Gao et al. [18] utilized phosphogypsum as a sulfate activator for SSC, demonstrating that phosphogypsum can be used to prepare environmentally friendly and high-performance SSC. The chemical composition and physical properties of these byproduct gypsums vary significantly depending on their source. In order to reduce the effect of instability in industrial by-product gypsum on SSC, the use of building gypsum (BG) can be considered as a sulfate activator for SSC. Magnesium oxide (MgO), recognized as an effective alkaline activator, has garnered significant attention in recent years due to its outstanding mechanical properties and durability [19,20,21,22]. In other words, MgO has received significant attention as an effective alkaline activator commonly employed in the activation process of GBFS. Compared to alkaline earth metals, alkali metals have a more significant activation ability for abrasive GBFS [23,24]. In addition, the environmental impact of alkali metal-activated GBFS is higher than that of alkaline earth metal-activated GBFS [25,26]. Among these, MgO is more effective at activating GBFS and yields higher compressive strength [21]. Active MgO is recommended as the preferred and effective activator for GBFS. However, research on the joint activation of GBFS by MgO and BG for stabilizing clay remains relatively limited. Otherwise, SSC concrete has micro-expansion properties and excellent resistance to alkali aggregate reaction and sulfate erosion. Whether the use of SSC solidified clay also has the ability to resist sulfate erosion still requires further research.

When solidified clay is employed as an engineering soil, the effects of environmental factors such as prolonged rainfall and temperature extremes can lead to changes in its mechanical properties [27,28]. Otherwise, in coastal areas, in the presence of SO_4_^2−^ and metal cations (e.g., sodium), solidified clay can be attacked by sulfates, leading to the accumulation of expansive gel substances (e.g., AFt crystals) within solidified clay. This process causes changes in particle position and volume deformation, which ultimately leads to a reduction in the strength and durability of solidified clay [29,30,31,32]. By utilizing the hydration of binder materials to pre-generate AFt crystals in solidified clay, the substances that react with external SO_4_^2−^ in the matrix can be significantly reduced [33,34]. Overall, solidified clay is affected by dry–wet cycles or attacks by sulfate solutions, all of which can impact the bonding between particles. The products generated by the hydration of the binder material are relatively single; the single gelling substance tends to become unstable in the presence of temperature fluctuations or water. If the types of gelling substances generated by the hydration of the binder materials are diverse and these gelling substances are interconnected, they may more effectively resist the adverse effects of temperature fluctuations or water. Currently, research on the application of SSC composed of GBFS, MgO, and BG in clay solidification is still limited, particularly with respect to the effects of dry–wet cycles and erosion from sodium sulfate solutions.

This work aims to explore and optimize the synergistic effects of SSC, which includes GBFS, MgO, and BG, in the solidification of soft clay. In this paper, first of all, the effect of synergistic interaction between GBFS, MgO, and BG on stabilized clay was investigated in terms of unconfined compressive strength (UCS) and splitting tensile strength (STS). Furthermore, based on the higher strength values of solidified clay, the optimum mix ratio of GBFS, MgO, and BG was determined. Subsequently, the dry–wet cycle performance of solidified clay and its attack performance in sodium sulfate solution were evaluated. Last but not least, the synergistic effects between GBFS, MgO, and BG on the types of hydration products generated from stabilized clay were analyzed through X-ray diffraction (XRD), scanning electron microscopy (SEM), Fourier transform infrared spectroscopy (FTIR), and thermogravimetric analysis (TGA), as well as the effects of sodium sulfate solution on the types and morphology of hydration products of solidified clay. This study provides important references for the application of SSC in the solidification of soft clay.

## 2. Materials and Methods

### 2.1. Materials

The lacustrine soft clay (SC) was derived from a construction site in Hangzhou, Zhejiang Province, China. The basic physical properties of the SC are presented in Table 1, in accordance with relevant standards [35,36,37]. According to ASTM D2974-14 [38], the loss on ignition (LOI) of the soft clay was measured to be 4.6%, which can be used to characterize the organic matter content.

**Figure 1:**
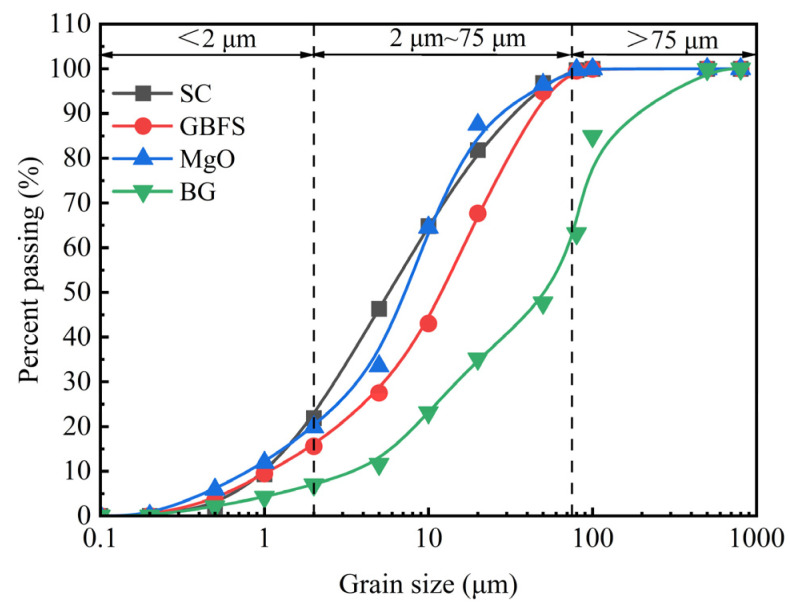
Particle size distribution curves of SC, GBFS, MgO, and BG.

The materials utilized for solidifying clay included GBFS, MgO, BG, and OPC. GBFS and MgO were purchased from Lingshou County Shiyun Mining Products Co., Ltd., which is located in Shijiazhuang City, Hebei Province, China. The 42.5# OPC and BG were purchased from the Building Materials Market, which is located in Xi’an, Shaanxi Province, China. In this study, the OPC met the requirements set forth by the Chinese test standard GB 175-2023 [39], as illustrated in Table 2. The grade of GBFS belongs to S105, according to the Chinese standard GB/T 18046-2017 [40]. The MgO used in this study was of chemical purity, with a purity of 98%. The LOI of GBFS, MgO, and BG was 2.85%, 2.0%, and 13.5%, respectively. The LOI of cement is shown in Table 2. The LOI of these four materials refers to the mass lost during the heating process. This indicator is mainly used to assess the content of volatile components in the materials, such as carbonates or moisture. The particle size distributions of SC, GBFS, MgO, and BG were measured with a Bettersize 2000LD laser particle size distribution meter (Dandong Baite Instrument Co., Ltd., Dandong, Liaoning, China), as shown in Figure 1. According to the particle size analysis curves, the particle size range of GMBF, MgO, and BG was mainly concentrated in the range of 2 μm~75 μm. The physical properties of GBFS, MgO, and BG are presented in Table 3. Based on the analysis of the particle size distribution and specific surface area of GMBF, MgO, and BG, the difference in the particle size of the materials has a positive effect on filling the pores. In addition, a larger specific surface area contributes to the reactivity of the materials. The determination of the pH of the raw material was aimed at clarifying the trend of the pH of the material after it was combined with the clay. The major chemical compositions of SC, GBFS, MgO, and BG tested by an X-ray fluorescence spectrometer are shown in Table 4. The chemical composition of SC primarily comprised Al_2_O_3_ and SiO_2_. The main chemical components of GBFS, MgO, and BG were CaO, MgO, and SO_3_, respectively. CaO was released mainly as Ca^2+^, while MgO released OH^−^ during hydration. BG generated SO_4_^2−^ during hydration. These ions interact with one another during the hydration reaction, resulting in the formation of a gel substance [11,13]. The chemical compositions of OPC and GBFS contained similar amounts of CaO and Al_2_O_3_. Research shows that GBFS has potential activity, but unlike OPC, GBFS cannot directly undergo hydration reactions; instead, it requires alkaline substances or sulfate materials to activate its activity [41].

**Figure 2:**
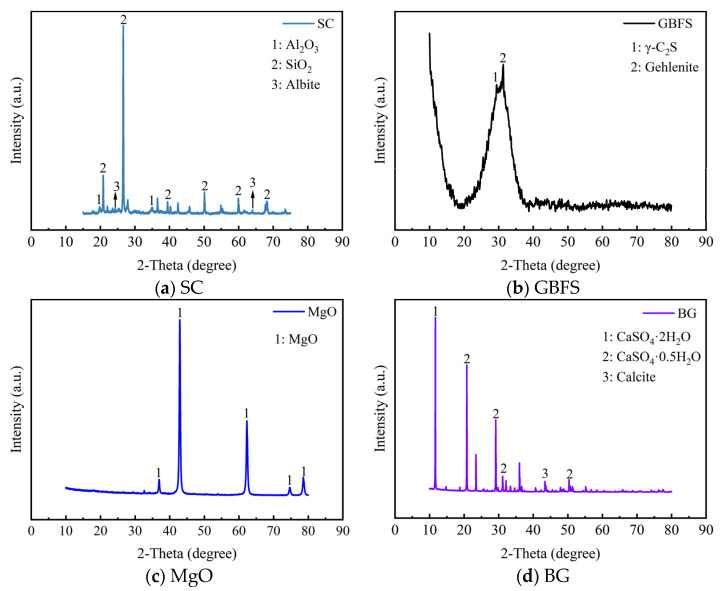
Phase analysis of the (**a**) SC, (**b**) GBFS, (**c**) MgO, and (**d**) BG.

**Figure 3:**
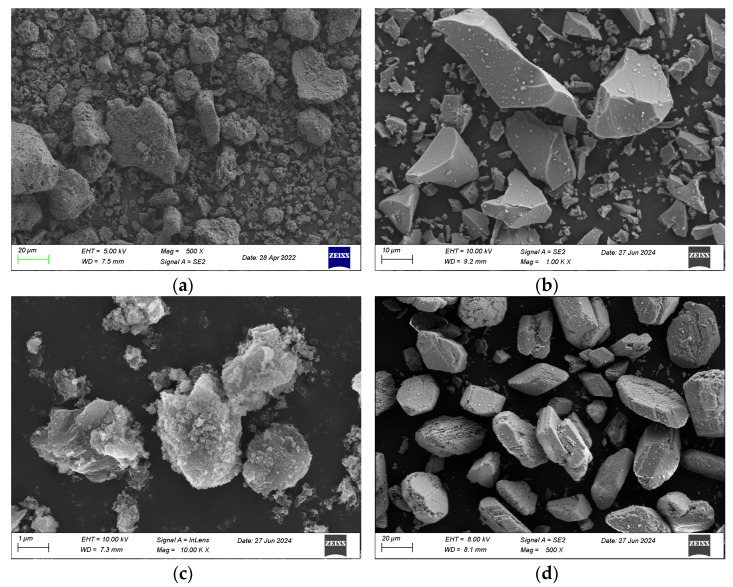
Micromorphology of (**a**) SC, (**b**) GBFS, (**c**) MgO, and (**d**) BG.

The mineral compositions of SC, GBFS, MgO, and BG tested by XRD are illustrated in Figure 2. The XRD pattern of the clay revealed the presence of SiO_2_ and Al_2_O_3_. The phase composition of the GBFS was mainly vitreous and contained a minor amount of gehlenite and γ-C_2_S [14]. The strong and sharp peak at 2*θ* = 42.9° indicated the high crystallinity of the MgO phase. In BG, CaSO_4_·0.5H_2_O and CaSO_4_·2H_2_O were found to be the main crystal phases. The microphotograph taken by SEM of SC, GBFS, MgO, and BG is illustrated in Figure 3. Soft clay particles exhibited irregular shapes, demonstrating a diverse particle distribution. The surfaces of the particles were comparatively rough and exhibited numerous tiny pores. GBFS particles exhibited an irregular shape yet possessed a smooth surface. MgO particles exhibited an oval shape characterized by an uneven and rough surface. BG particles exhibited a rectangular shape and featured micropores on their surface.

### 2.2. Design of Binder Materials and Sample Preparation

SSC typically consists of about 70–90% blast furnace slag, 10–20% calcium sulfate, and 5–15% alkaline activator [11,12,13,16]. When used as soft clay curing, the binder materials typically constitute 10% or 15% of the dry soil weight [42]. The binder materials used in this experiment consist of GBFS, MgO, and BG, with their contents shown in Table 5. For instance, the binder material A1 was mixed with clay to form S-A1 solidified clay. It is important to note that S-A2 and S-B0 belong to the same experimental group. In fact, the results of the experiments in the two groups were consistent. Therefore, S-A2, as described later, can be considered S-B0.

**Figure 4:**
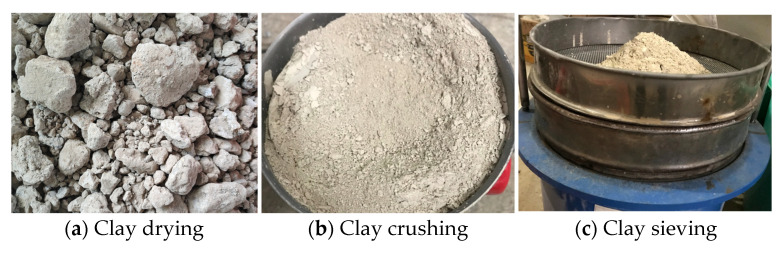
Treatment process of clay.

The preparation and curing of the samples were conducted according to the following steps:

(I) The lacustrine soft clay was dried and passed through a 2 mm sieve to remove impurities, and the treatment process is shown in Figure 4.

(II) To simulate the mixing process of binder materials and soft clay in the foundation, distilled water was added to the dry clay and then mixed using a mechanical mixer, with the moisture content set to 70%. The premixed clay was then stored in an airtight container for 24 h to ensure an even distribution of moisture content.

(III) The binary binder materials (GBFS and MgO) or ternary binder materials (GBFS, MgO, and BG) were evenly mixed in predetermined proportions before being added to clay at a fixed ratio of 10% or 15% by dry mass of soil. Similarly, cement was used as a binder for soft clay as a control group.

(IV) The mixed paste was filled into the PVC cylindrical mold in 5–6 layers. After adding each layer of the mixture in the mold, a vibrating device was used to vibrate for 5–10 min to remove bubbles from the sample. Then, the next layer was added until the mold was completely filled. PVC cylindrical molds with an inner diameter of 39.1 mm and a height of 80 mm were used for the UCS test, while molds with an inner diameter and height of 50 mm were used for the STS test.

(V) The surface of the samples was covered with plastic wrap, and the molds were demolded after 24 h of curing in a curing box. Subsequently, the samples were encapsulated in plastic bags and placed in a curing box for curing until the required age was reached. The temperature was controlled at 20 °C ± 2 °C, and the humidity was maintained above 96%.

### 2.3. Testing Procedures

#### 2.3.1. Test Method for Unconfined Compressive Strength

The test method for UCS was based on the T0805-2024 standard of JTG 3441-2024 [43]. A WDW-100D electronic universal testing machine(Shenzhen Sunway Technology Co., Ltd., Shenzhen, Guangdong, China) was utilized, featuring a maximum loading capacity of 20 kN. The strain-controlled loading rate was set at 1 mm/min. The equation used to calculate the UCS (*R_c_*) is provided below:

(1) $$R_{c}=P/A=4P/\pi D^{2}$$

where *R_c_* is the UCS (MPa); *A* is the cross-sectional area of the sample (mm^2^); *P* is the maximum pressure when the sample is damaged (N); and *D* is the diameter of the sample (mm). Each group consisted of three sample replicates, and the final UCS was determined from the average strength of these three samples.

#### 2.3.2. Test Method for Splitting Tensile Strength

The test method for the STS was based on the T0808-1994 standard in JTG 3441-2024 [43]. A WDW-100D electronic universal testing machine was utilized, featuring a maximum loading capacity of 20 kN. The strain-controlled loading rate was set at 1 mm/min. The equation used to calculate the STS (*R_i_*) is provided below:

(2) $$R_{i}=2P/\pi Dh$$

where *R_i_* is the STS (MPa); *h* is the height of the sample (mm); *P* is the maximum pressure when the sample is damaged (N); and *D* is the diameter of the sample (mm). Each group consisted of three sample replicates, and the final STS was determined from the average strength of these three samples.

#### 2.3.3. Dry and Wet Cycle Test

In coastal areas, when utilizing solidified clay as general foundation fill, it was essential to consider the influence of groundwater on the soil. The fluctuation of groundwater causes water molecules to repeatedly enter and exit the soil [27,28]. These cycles of drying and water absorption affected the connections between soil particles. The dry–wet cycle test aims to simulate the repeated processes of dewatering and water absorption in the soil, allowing for an evaluation of the changes in the physical and mechanical properties of solidified clay.

According to the wet and dry cycle test method of ASTM D4843-88 [44], the samples were cured for 28 days and 91 days, respectively, in order to perform the wet and dry cycle test. The detailed wet and dry cycle was as follows: (I) the sample was soaked in pure water for 24 h; (II) the sample was dried in a drying oven at 45 °C for 24 h to achieve the purpose of removing free water.

#### 2.3.4. Sodium Sulfate Solution Soaking Test

In coastal areas, the use of solidified clay for foundations may be exposed to sulfate attack, which can lead to compromised performance [45,46,47]. The degree of deterioration may vary significantly depending on the composition of the binder [48]. Therefore, it was essential to assess the sulfate erosion resistance of solidified clays.

To assess sulfate resistance, samples cured for 91 days were soaked in a Na_2_SO_4_ solution for 1, 3, 7, 14, and 28 days. In addition, it should be emphasized that the Na_2_SO_4_ mass fraction in the solution was 5%. Finally, UCS tests and pH measurements were conducted on these samples.

#### 2.3.5. pH Measurements

According to ASTM D4972-19, the pH value of solidified clay, after soaking in sodium sulfate solution, was measured [49]. Representative fragments from the UCS test samples were ground and dried in an oven at 40 °C, then passed through a 2 mm sieve. Subsequently, 10 g of the sieved powder was mixed with 50 mL of distilled water in a beaker, stirred for 5 min, and allowed to stand for 60 min. Finally, the pH value of the suspension solution was measured with a pH meter.

#### 2.3.6. Mineral and Microstructural Analyses

Before analyzing the microstructure and mineral phases, representative fragments from UCS test samples were soaked in anhydrous ethanol for 24 h. After soaking, they were dried at a temperature of 50 °C. To investigate the mineral phases, the dried fragments were ground and sieved through a 200-mesh sieve (0.074 mm) before being scanned using an Rigaku SmartLab SE X-ray diffractometer equipped with a Cu Kα source, covering a range from 15° to 75° with a step size of 0.02°. The Rigaku SmartLab SE X-ray diffractometer used in this study is manufactured by Rigaku Corporation, which is located in Tokyo Metropolis, Japan. The dried fragments were coated with a thin layer of gold and then analyzed using a Sigma 300 instrument to identify the hydrated phases and micromorphology. The Sigma 300 instrument used in this study is manufactured by Carl Zeiss AG, which is located in Oberkochen, Germany. TGA tests were conducted over a temperature range of 30 °C to 1000 °C, at a heating rate of 10 °C/min in a nitrogen atmosphere. FTIR tests were performed using the potassium bromide pellet method, with 32 scans at a resolution of 4 cm^−1^ over a wavenumber range of 400–4000 cm^−1^.

In summary, the test designs for mechanical properties and microscopic tests are shown in Table 6 and Table 7. N-(S-A2) indicates that S-A2 has been soaked in sodium sulfate solution, while N-(S-B1) indicates that S-B1 has been soaked in sodium sulfate solution.

## 3. Results and Discussion

### 3.1. UCS and STS of Solidified Clay

**Figure 5:**
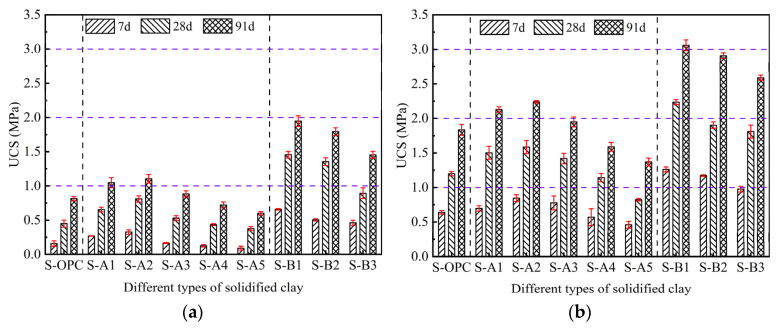
Relationship between different GBFS, MgO and BF contents and UCS of solidified clay. (**a**) The binder materials account for 10% of the dry clay mass. (**b**) The binder materials account for 15% of the dry clay mass.

The UCS of solidified clay with varying proportions of GBFS, MgO, and BG is shown in Figure 5. The UCS of all samples significantly increased with the extension of the curing time. The UCS of each sample increased significantly as the dosage of binder material increased from 10% to 15%. When the MgO content was 20%, the UCS fluctuated sharply with the content of GBFS or BG. Specifically, the UCS of the sample decreases with the reduction in GBFS content, while it also decreases with the increase in BG content. However, as the MgO content increased from 10% to 50%, the UCS of the samples first increased and then decreased. Specifically, the UCS of S-A2 was higher than that of S-A1, S-A3, S-A4, and S-A5 at 7, 28, and 91 days. As the MgO content increased from 10% to 50%, the corresponding GBFS content gradually decreased, and the GBFS provided insufficient active material (alumina), which led to a decrease in the UCS of the sample with decreased GBFS content. At this time, the UCS of S-A5 was lower than that of the control S-OPC, and the UCS of S-A5 was the lowest value. This indicates that an excess of alkaline substances used to activate slag may have negative effects. When the hydration of the alkaline substance MgO creates an alkaline environment, it does not result in a higher UCS for solidified clay. The UCS of S-A1, S-A2, S-A3, S-A4, and S-A5 was lower than that of S-B1, S-B2, and S-B3 at 7, 28, and 91 days. It can be seen that the hydration of MgO, along with the hydration of BG, produced an alkaline environment, both stimulating the reactive activity of GBFS. The UCS of S-B2 solidified clay was the highest among all samples when the MgO content was set at 20% and the BG content reached 20%. As the BG content increased from 20% to 60%, the UCS of solidified clay gradually decreased. In summary, excessively high contents of either MgO or BG were not conducive to strength improvement.

**Figure 6:**
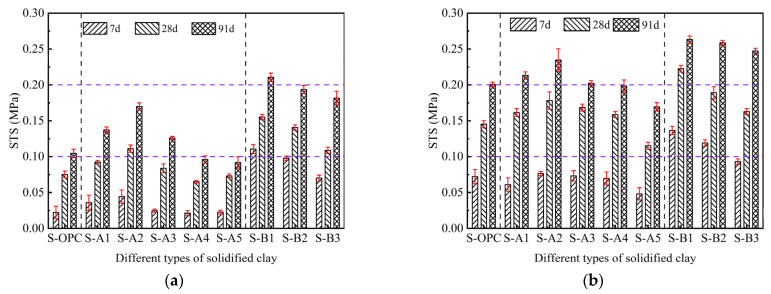
Relationship between different GBFS, MgO, and BF contents and STS of solidified clay. (**a**) The binder materials account for 10% of the dry clay mass. (**b**) The binder materials account for 15% of the dry clay mass.

The STS of solidified clay with different ratios of GBFS, MgO, and BG is shown in Figure 6. The STS of each sample increased significantly with the extension of the curing time. Meanwhile, the STS of each sample also increased significantly as the binder dosage increased from 10% to 15%. The variation in STS exhibited a similar trend to that of UCS. Based on the evaluation of the samples presented in Figure 5 and Figure 6, when the binary binder materials (GBFS and MgO) constitute 10% or 15% of the dry clay mass, S-A2 demonstrates exceptional UCS and STS, with the optimal mass ratio of GBFS to MgO being 8:2. Similarly, when the ternary binder materials (GBFS, MgO, and BG) constitute 10% or 15% of the dry clay mass, S-B1 demonstrates exceptional UCS and STS, with the optimal mass ratio of GBFS, MgO, and BG being 6:2:2.

Figure 5 and Figure 6 illustrate the effectiveness of binary binder materials (GBFS and MgO) and ternary binder materials (GBFS, MgO, and BG) on the UCS and STS of soft clay. The effect of binary binder materials on solidified clay was weaker than that of ternary binder materials. In addition, in the clay solidified with GBFS and MgO, the UCS and STS of S-A4 and S-A5 were both lower than those of the S-OPC, while the UCS and STS of S-A1, S-A2, and S-A3 were higher than those of the S-OPC. In the clay solidified with GBFS, MgO, and BG, the UCS and STS of S-B1, S-B2, and S-B3 were higher than those of the S-OPC. When the proportion of binder materials constitutes 10% of the dry clay mass, the UCS of S-B1 increases by 101.81%, 79.65%, and 76.4% compared to S-A2 at curing ages of 7 days, 28 days, and 91 days, respectively. Similarly, when the binder materials account for 15% of the dry clay mass, the UCS of S-B1 shows increases of 49.28%, 41.02%, and 36.55% compared to S-A2 at the same curing ages. There was a more significant role of BG compared to GBFS, as the enhancement effect of BG primarily occurred in the early stage (7 days). These results indicated that the use of ternary binder materials with solidified clay was feasible and exhibited superior strength properties compared to the use of pure cement-solidified clay. Although binary binders have the ability to improve the properties of soft clay, they produce a relatively limited variety of gel substances. In S-A5, MgO comprised 50% of the mass of the binder materials, and the gel substances produced by its hydration were expansive, leading to instability in the solidified soft clay system. As the mass of BG increased, the UCS and STS of S-B1 to S-B3 exhibited a decreasing trend, primarily because the crystals produced by the hydration of BG initially filled the pore spaces of solidified clay. When the pore space could no longer accommodate additional crystals, the expansive nature of these crystals disrupted the inter-particle linkage. This explains the lower UCS and STS of S-B3 compared to S-B1.

**Figure 7:**
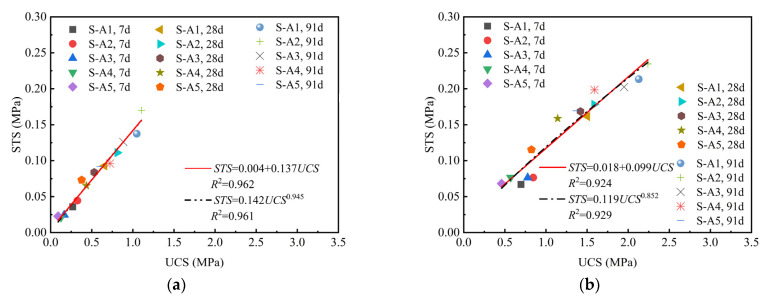
The relationship between UCS and STS of binary binder solidified clay. (**a**) The binder materials account for 10% of the dry clay mass. (**b**) The binder materials account for 15% of the dry clay mass.

**Figure 8:**
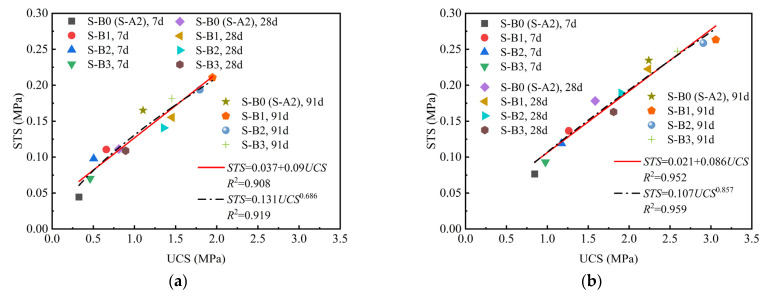
The relationship between UCS and STS of ternary binder solidified clay. (**a**) The binder materials account for 10% of the dry clay mass. (**b**) The binder materials account for 15% of the dry clay mass.

Several studies indicated that there was a linear relationship between the tensile strength and the compressive strength of cement soil [19,50,51]. Therefore, this study investigated the correlation between the STS and the UCS of both binary and ternary binder solidified clay. Figure 7 and Figure 8 present scatter diagrams illustrating the relationships between STS and UCS, based on the experimental data for binary and ternary binder solidified clay, respectively. Linear and power functions were applied to fit the experimental data in Figure 7 and Figure 8, with the corresponding results presented in the equations within the figure. Similarly, linear and power functions were applied to fit the experimental data in Figure 7 and Figure 8, with the corresponding results presented in the equations within the figure. As illustrated in Figure 7 and Figure 8, the STS of binary and ternary binder solidified clay increases with compressive strength. There existed a strong linear relationship between the two.

### 3.2. Durability of Solidified Clay

In binary-bonded solidified clay, the mass ratio of GBFS to MgO was 8:2. In ternary bonded solidified clay, the mass ratio of GBFS, MgO, and BG was 6:2:2. These compositions contribute to the high UCS observed in solidified clay. At the curing time of 28 days, the UCS of S-A2 and S-B1 was 1.59 MPa and 2.236 MPa, respectively. At 91 days of curing, the UCS of S-A2 and S-B1 increased to 2.24 MPa and 3.059 MPa, respectively. This indicated that the UCS of solidified clay met the application requirements of relevant engineering. To further evaluate the durability performance of solidified clay, dry–wet cycle and sodium sulfate solution soaking tests were conducted for binary and ternary bonded solidified soft clay to clarify the differences between S-A2 and S-B1 under dry–wet cycle and sodium sulfate solution soaking conditions.

#### 3.2.1. Dry–Wet Cycle of Solidified Clay

**Figure 9:**
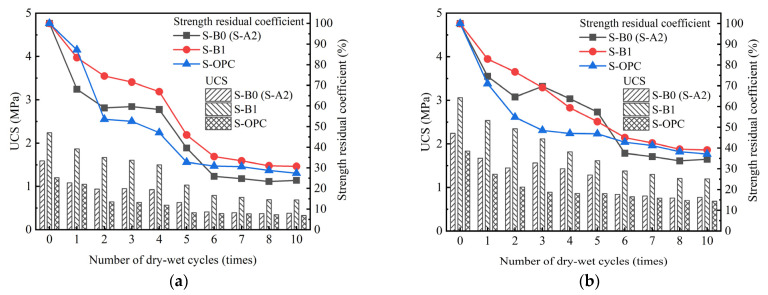
Effect of dry–wet cycle on the UCS of solidified clay at (**a**) 28 days and (**b**) 91 days.

The variation patterns of the UCS for S-A2, S-B1, and S-OPC with respect to the number of wet–dry cycles are illustrated in Figure 9.

The trend depicted in the line graph in Figure 9 indicates that the UCS development of S-A2, S-B1, and S-OPC can be categorized into two phases: a steep decline, representing a rapid decay of UCS, and a gentle decline, indicating a slow decay of UCS, with the sixth wet–dry cycle serving as the dividing point. When the number of wet–dry cycles ranged from one to six, the UCS of S-A2 and S-B1 exceeded that of S-OPC. The strength residual coefficient is defined as the ratio of the strength of solidified clay following each wet–dry cycle to its initial strength. In Figure 9a, after six wet–dry cycles, the strength residual coefficient of S-A2, S-B1, and S-OPC was 25.87%, 35.44%, and 30.89%, respectively. In Figure 9b, after six wet–dry cycles, the strength residual coefficient of S-A2, S-B1, and S-OPC was 37.47%, 45.05%, and 42.78%, respectively. It is worth noting that relevant studies indicate that the UCS of solidified clay significantly decreases after experiencing the first wet–dry cycle [52,53]. During six to ten wet–dry cycles, the UCS of S-A2 was lower than that of S-OPC, whereas the UCS of S-B1 was higher than that of S-OPC. It can be said that extended curing time helps to ameliorate the damage caused by wet–dry cycles to solidified clay.

The dry–wet cycle can lead to the deterioration of the properties of solidified clay, mainly for the following reasons [53,54]: (I) During the wet–dry cycling process, free water repeatedly entered and exited solidified clay, causing its internal materials to continue reacting. Unreacted magnesium oxide existed in solidified clay. It reacted with water to form magnesium hydroxide, which subsequently lost moisture under dry conditions and transformed back into magnesium oxide crystals. BG contains CaSO_4_·0.5H_2_O and CaSO_4_·2H_2_O. Unreacted CaSO_4_·0.5H_2_O existed in solidified clay; its conversion to CaSO_4_·2H_2_O led to changes in particle positioning, thereby affecting the overall stability of solidified clay. These reactions caused microcracks and pores to appear on the surface of solidified clay, leading to a reduction in UCS, thereby decreasing the performance of solidified clay. (II) During the drying process, moisture in solidified clay continuously evaporates. The rate of moisture loss from the surface was higher than that from the interior, resulting in internal pressure within solidified clay while the surface experiences tensile forces. When the surface of solidified clay experienced significant tension, microcracks developed on it. (III) During the wet–dry cycling process, free water infiltrated solidified clay, causing its volume to undergo slight changes due to water absorption expansion. When the stress generated by the deformation of solidified clay exceeded its structural strength, it concentrated at the weak connections between particles, leading to the formation of microcracks.

#### 3.2.2. Sulfate Attack Resistance of Solidified Clay

**Figure 10:**
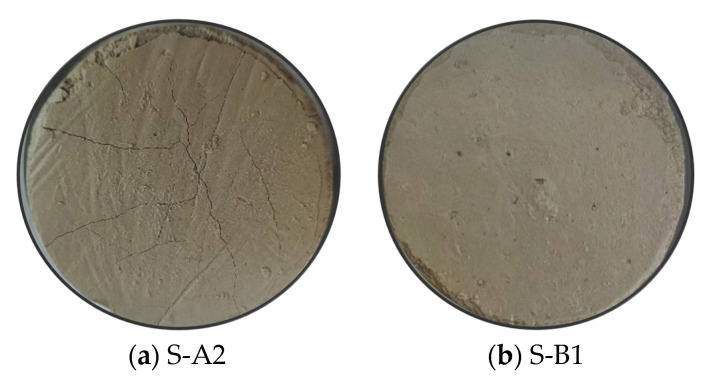
Appearance of solidified clay after 28 days of soaking in sodium sulfate solution.

Figure 10 shows the appearance of S-A2 and S-B1 after being soaked in sodium sulfate solution for 28 days. After soaking in sodium sulfate solution for 28 days, the top surface of the S-A2 sample exhibited microcracks, whereas the top surface of the S-B1 sample exhibited no microcracks. Microcracks on the top surface of the S-A2 sample resulted in structural loosening, and these cracks may extend into the interior of solidified clay, indicating that the overall structural integrity was compromised. In contrast, although the top surface of the S-B1 sample did not exhibit microcracks, the sodium sulfate solution still exerted a notable attack effect on it. To further verify the effect of the sodium sulfate solution on solidified clay, it was evaluated by performing UCS.

**Figure 11:**
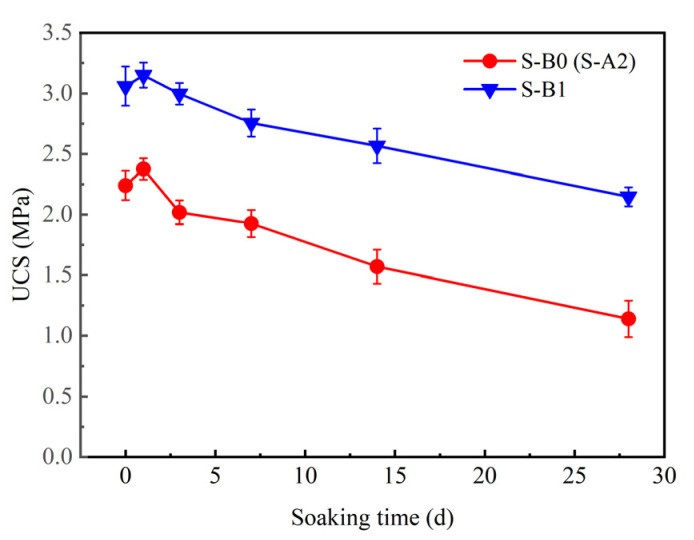
Relationship between the UCS of solidified clay and soaking time.

Figure 11 illustrates the relationship between the UCS of solidified clay and soaking time. After curing solidified clay for 91 days, the UCS of S-A2 and S-B1 were 2.24 and 3.059 MPa, respectively. After soaking solidified clay in sodium sulfate solution for one day, the UCS of S-A2 and S-B1 increased by 6% and 3%, respectively, compared to their values prior to soaking. After soaking solidified clay in sodium sulfate solution for 28 days, the UCS of S-A2 and S-B1 decreased by 1.1 and 0.89 MPa, respectively. The UCS of S-A2 and S-B1 decreased by 49.1% and 29.8%, respectively, compared to their values before soaking. This deterioration results from the coupled effects of water and SO_4_^2−^. After solidified clay was soaked in sodium sulfate solution for one day, the UCS of S-A2 and S-B1 increased, which can be attributed to the formation of newly developed AFt (3CaO·Al_2_O_3_·3CaSO_4_·32H_2_O) crystals on the surface of solidified clay, as expressed by Equations (3) and (4) [55,56]. The influence of endogenous and exogenous AFt crystals was considered a key factor causing this difference. The exogenous AFt crystals were newly generated from the external SO_4_^2−^ and the substances in solidified clay (Equations (3) and (4)), whereas endogenous AFt crystals primarily originated from the existing AFt crystals in S-B1 (Equations (5) and (6)) [57]. The formation of AFt crystals helps to improve the compactness of solidified clay, especially in the early stages of solidification [58,59,60,61]. On the other hand, AFt crystals usually maintain stability in sulfate solutions, which may help improve the sulfate resistance of solidified clay. As the crystals expand, they preferentially occupy the pores on the surface of solidified clay. However, due to structural limitations beneath the surface of solidified clay, the forces generated by the expansion of the newly formed crystals within solidified clay may initiate internal microcracks. It is important to note that S-B1 originally contained AFt crystals, resulting in a relatively low concentration of free Ca^2+^ in the system. Consequently, the introduction of exogenous SO_4_^2−^ into S-B1 led to the formation of fewer newly developed crystals. In addition, the sodium sulfate solution influences C-S-H gel in both S-A2 and S-B1. The presence of sodium sulfate leads to a loss of Ca^2+^ from C-S-H gel [62], which subsequently affects the UCS of S-A2. However, in S-B1, the overall structure formed by the close combination of AFt crystals and C-S-H gel can effectively resist this kind of erosion.

(3) $$Na_{2}SO_{4}+Ca{\left(OH\right)}_{2}+2H_{2}O\to CaSO_{4}\cdot 2H_{2}O+2NaOH$$

(4) $$Al_{2}O_{3}+3Ca{\left(OH\right)}_{2}+23H_{2}O+3CaSO_{4}\cdot 2H_{2}O\to 3CaO\cdot Al_{2}O_{3}\cdot 3CaSO_{4}\cdot 32H_{2}O$$

(5) $$3CaO\cdot Al_{2}O_{3}+Ca{\left(OH\right)}_{2}+12H_{2}O\to C_{4}AH_{13}$$

(6) $$C_{4}AH_{13}+3(C\mathrm{aS}O_{4}\cdot 2H_{2}O)+14H_{2}O\to 3CaO\cdot Al_{2}O_{3}\cdot 3CaSO_{4}\cdot 32H_{2}O+Ca{\left(OH\right)}_{2}$$

**Figure 12:**
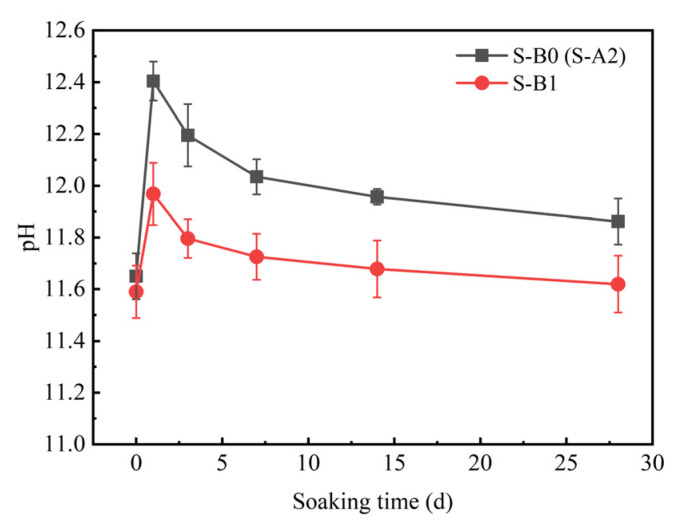
Relationship between the pH value of solidified clay and soaking time.

Figure 12 shows the relationship between the pH of solidified clay and soaking time. After 91 days of curing solidified clay, the pH values of S-A2 and S-B1 were 11.65 and 11.59, respectively. In comparison to S-A2, S-B1 exhibited a lower pH of 11.58, resulting from the partial replacement of GBFS with BG. In the case of S-B2, hydroxide ions (OH^−^) were required to participate in the hydration reactions of GBFS and BG, and thus OH^−^ was consumed. The pH values of S-A2 and S-B1 increased by 6.5% and 3.3%, respectively, from their initial values after solidified clay was soaked in a sodium sulfate solution for one day. The increase in pH accelerated the hydration reaction, which was responsible for the generation of new crystals on the surface of solidified clay. Ca(OH)_2_ was consumed by the production of AFt crystals. S-B1 already contained CaSO_4_·2H_2_O, and the production of AFt crystals consumed Ca(OH)_2_ in the system, resulting in less available Ca(OH)_2_. In the presence of SO_4_^2−^, only a limited number of new AFt crystals were formed. Therefore, the sodium sulfate solution affected S-A2 to a greater extent than S-B1. In addition, the generation of AFt crystals required a pH of at least 10 in the liquid phase, and a higher pH was needed to ensure its stability [63,64,65]. During the process of soaking solidified clay in sodium sulfate solution, the pH values of S-A2 and S-B1 initially increased and then decreased. However, the final pH after soaking was still higher than the initial value. This also indicated that the soaking process of sodium sulfate solution has less effect on AFt crystals. This explains that the binding of C-S-H gel with AFt crystals in S-B1 may mitigate the erosion of hydrated calcium silicate.

## 4. Microscopic Results

### 4.1. XRD of Binder Materials

**Figure 13:**
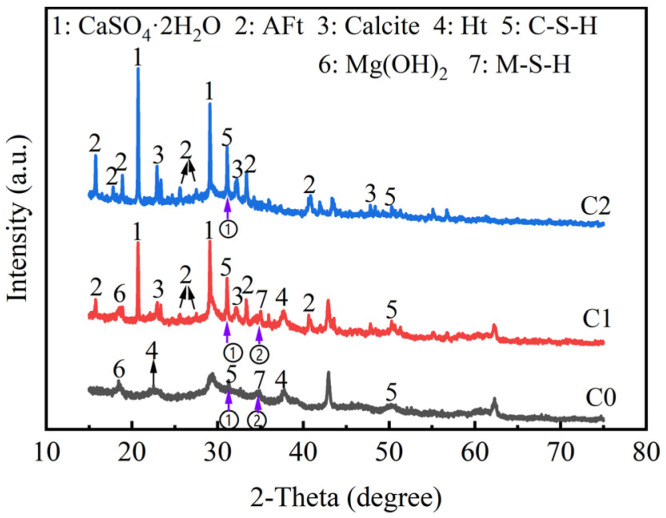
XRD patterns of C0, C1, and C2 binder materials.

To more clearly identify the mineral phases of the binder materials, three types of pastes were designed, and their phase composition was determined by XRD. The three types of pastes were as follows: the mass ratio of GBFS to MgO was 8:2 (C0), the mass ratio of GBFS, MgO, and BG was 6:2:2 (C1), and the mass ratio of GBFS to BG was 8:2 (C2). It was worth noting that the water–binder ratios of all three pastes were 0.35. The XRD results of the three types of pastes after 91 days of curing are shown in Figure 13.

The gel substance of the C1 pastes primarily consisted of AFt, CaSO_4_·2H_2_O, and C-S-H gel. At the marked position ① in Figure 13, the characteristic diffraction peaks of C-S-H for both C1 and C2 appear at *2θ* of around 30° [66]. At the indicated position ② in Figure 13, magnesium silicate hydrate (M-S-H) was present only in C0 and C1. MgO contributes to an alkaline environment and provides Mg^2+^ necessary for the formation of M-S-H. The characteristic diffraction peaks of AFt were most pronounced in C2, followed by C1, while its diffraction peaks were not observed in C0. This indicated that BG, as a sulfate activator, easily formed AFt when combined with GBFS [67]. Compared to C2, C1 contains Mg(OH)_2_ and hydrotalcite-like (Ht) gel. It was worth noting that the intensity of the characteristic diffraction peaks of AFt was correlated with the characteristic diffraction peaks of CaSO_4_·2H_2_O. For instance, the characteristic diffraction peak of CaSO_4_·2H_2_O in C1 was relatively weak, while the diffraction peak in C2 was the most pronounced, at 2*θ* of 11.68°, 20.86°, and 29.2°. The mass of BG in the pastes of C1 and C2 was consistent, comprising 20% of the total composition. In C1, MgO acted as an alkaline activator, contributing to a higher involvement of BG in the hydration reaction, which led to the conversion of CaSO_4_·2H_2_O to other gel substances.

### 4.2. XRD of Solidified Clay

**Figure 14:**
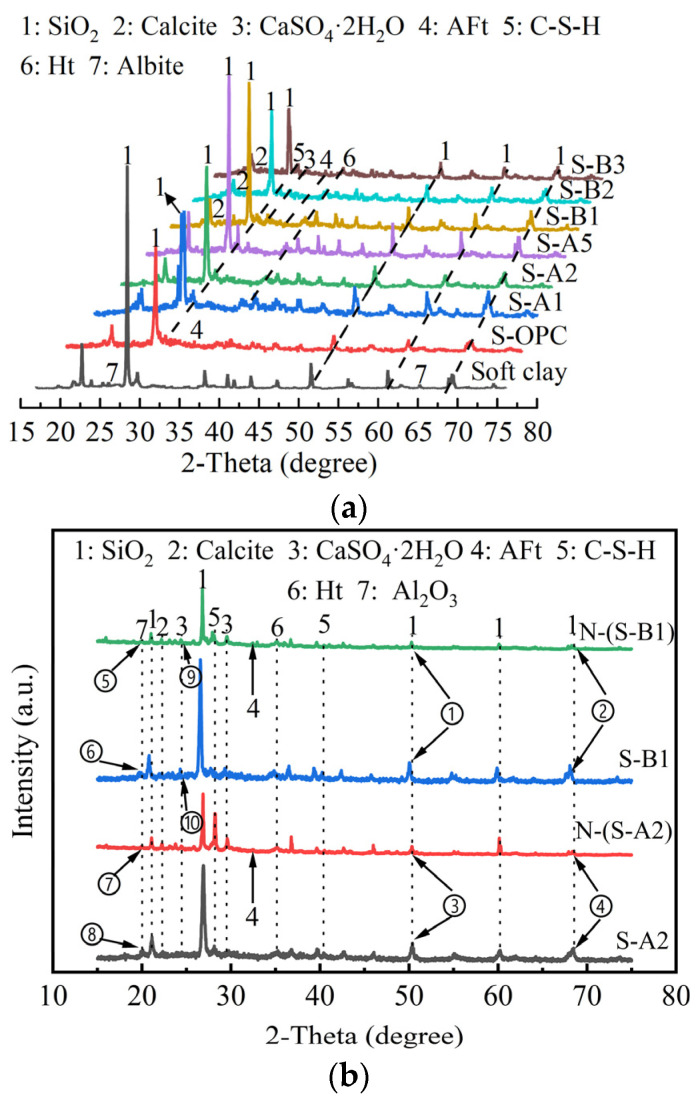
XRD patterns of solidified clay. (**a**) XRD patterns of different binder materials stabilized clay after 91 days of curing. (**b**) XRD patterns of solidified clay soaked in sodium sulfate solution for 28 days.

The XRD patterns of solidified clay are presented in Figure 14. The XRD patterns for solidified clay after 91 days of curing are presented in Figure 14a. Additionally, the XRD patterns for solidified clay soaked in sodium sulfate solution for 28 days are shown in Figure 14b.

The diffraction peaks of quartz and albite observed in SC primarily reflected its mineralogical composition. MgO was identified in S-A1, S-A2, S-A5, S-B1, S-B2, and S-B3, with its characteristic peaks observed at 2*θ* = 36.3° and 43°. MgO was crucial for the formation of Ht gel [68,69]. S-A5 produces a greater amount of Ht compared to S-A2, as indicated by the diffraction angles of 2*θ* = 34.7° and 52° [70]. The presence of excess Ht in solidified clay adversely affected its structure due to the expansion properties of Ht, which influenced the UCS of solidified clay. Therefore, the UCS of S-A5 was lower than that of S-A2 due to this factor (see Figure 5). In addition, Ca^2+^ reacted with the Si-O and Al-O bonds in the GBFS to form C-S-H gel, which showed diffraction peaks in the range of 2*θ* = 26°~29° [71]. In S-B1, the co-presence of Al_2_O_3_ and SO_3_ promoted its main conversion to AFt. It is worth noting that the presence of AFt was also detected in S-B2 and S-B3.

Quartz, calcite, Ht, and aluminum oxide (Al_2_O_3_) were detected in S-A2, S-B1, N-(S-A2), and N-(S-B1). Compared to S-A2, the diffraction intensity peak of quartz in N-(S-A2) has decreased. Similarly, in N-(S-B1), the diffraction intensity peak of quartz has also diminished relative to S-B1, as indicated by positions ①, ②, ③, and ④ in Figure 14b. This indicates that SO_4_^2−^ enters solidified clay, affecting the diffraction intensity of quartz, which can be observed through the height of the diffraction peaks. Compared to S-A2, the diffraction intensity peak of C-S-H gel in N-(S-A2) decreased. Similarly, in N-(S-B1), the diffraction intensity peak of C-S-H gel also diminished relative to S-B1. This further confirms that SO_4_^2−^ in sodium sulfate solution makes C-S-H gel unstable, leading to its susceptibility to losing Ca^2+^. The chemical composition of C-S-H gel contains a large amount of Ca^2+^. In sodium sulfate solution, SO_4_^2−^ can exchange ions with Ca^2+^ in C-S-H gel, generating soluble Na^+^ and insoluble CaSO_4_. This hydration reaction not only consumes Ca^2+^ in the gel but also leads to a phase change in C-S-H, forming new mineral phases. This phase change may make the microstructure of solidified clay more fragile, reducing its durability. At 2*θ* = 29.96°, C-S-H gels were detected in S-A2 and S-B1, whereas N-(S-A2) and N-(S-B1) did not exhibit any C-S-H gels. In addition, the diffraction intensity peak of Al_2_O_3_ in N-(S-A2) decreased compared to S-A2, and a similar decrease was observed in N-(S-B1) relative to S-B1, as illustrated by positions ⑤, ⑥, ⑦, and ⑧ in Figure 14b. This indicates that exogenous SO_4_^2−^ promotes the formation of AFt when combined with Al_2_O_3_. Moreover, CaSO_4_·2H_2_O was also detected in S-B1 and N-(S-B1), as indicated by positions ⑨ and ⑩ in Figure 14b. The presence of CaSO_4_·2H_2_O in N-(S-A2) and N-(S-B1) results from the reaction between exogenous SO_4_^2−^ and free Ca^2+^ in solidified clay.

### 4.3. SEM and EDS of Solidified Clay

**Figure 15:**
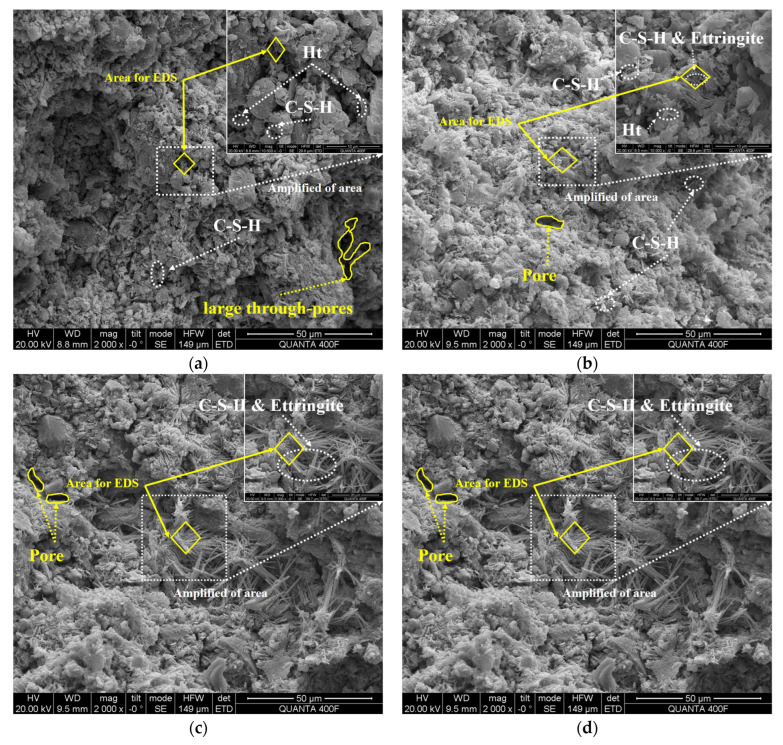
SEM images of (**a**) S-A2 and (**b**) S-B1 after 91 days of curing as well as (**c**) N-(S-A2) and (**d**) N-(S-B1) soaked in sodium sulfate solution for 28 days.

Figure 15 illustrates the typical sectional morphology of solidified clay as identified by SEM. In Figure 15a,b, the typical morphology of solidified clay after 91 days of curing is shown, with magnifications of 2000× and 10,000×, respectively. In Figure 15c,d, the typical morphology of solidified clay soaked in sodium sulfate solution for 28 days is shown, with magnifications of 2000× and 5000×, respectively.

The micrographs of solidified clay revealed the presence of gelled hydrates and AFt crystals. The primary distinctions between these gelled hydrates and AFt crystals were evident in their morphological and distributional characteristics [71]. The micrograph of S-A2 (Figure 15a) displayed needle-like C-S-H gel and flake-like Ht gel. The micrograph of S-B1 (Figure 15b) revealed the presence of needle-like C-S-H gel, flake-like Ht gel, needle-like CaSO_4_·2H_2_O, and AFt crystals. Compared to S-B1, S-A2 has relatively more pores or through pores. This is due to the fact that S-B1 contains more types of hydration products, which can fill the pores between particles, resulting in fewer pores. The hydration process of MgO provides an alkaline environment, and the combination of Al_2_O_3_ contained in GBFS and SO_3_ in BG promotes the growth of the expansive mineral AFt crystals. This explains the high mechanical strength of S-B1 compared to S-A2.

The micrographs of N-(S-A2) and N-(S-B1) (Figure 15c,d) revealed needle-like C-S-H gel and AFt crystals. In N-(S-A2), pores between particles were observed, accompanied by the formation of large through pores. N-(S-B1) exhibited pores between particles; however, no large through pores were present. A large amount of gelatinous substance was not observed in the pores of N-(S-A2); only a small amount of needle-like gel substance was present between the particles. A large amount of gelatinous substance was observed between the particles of N-(S-B1). These needle-like gel substances primarily consisted of C-S-H gel and AFt crystals, which interlocked to form agglomerates. Compared to S-A2, the needle-like substances in N-(S-A2) were reduced, indicating that the sodium sulfate solution erodes C-S-H gel, leading to the release of Ca^2+^. Free Ca^2+^ reacts with SO_4_^2−^ in the sodium sulfate solution to form CaSO_4_·2H_2_O. This CaSO_4_·2H_2_O subsequently combines with Al_2_O_3_ to produce AFt crystals, which were responsible for the formation of agglomerates in N-(S-A2). Compared to S-B1, the morphology of AFt crystals in N-(S-B1) has hardly changed, indicating that sodium sulfate solution does not alter its morphology. The morphology of AFt crystals in S-B1 was similar to that of N-(S-B1). These AFt crystals are interwoven and dispersed in the pores with C-S-H gel, which can be observed in Figure 15d. The newly formed AFt crystals aggregate closely intertwine with the existing AFt crystals in the previously solidified clay, resulting in a clustered pattern. These significantly elongated AFt crystals not only fill the pores but also preferentially provide structural support, serving as a framework and bridging adjacent components. The AFt crystals encapsulate the gel-like hydrates, thereby preventing erosion and contributing to a more robust structure. This observation also explains why N-(S-B1) exhibits superior mechanical properties compared to N-(S-A2). The specific strength changes are shown in Figure 10.

**Figure 16:**
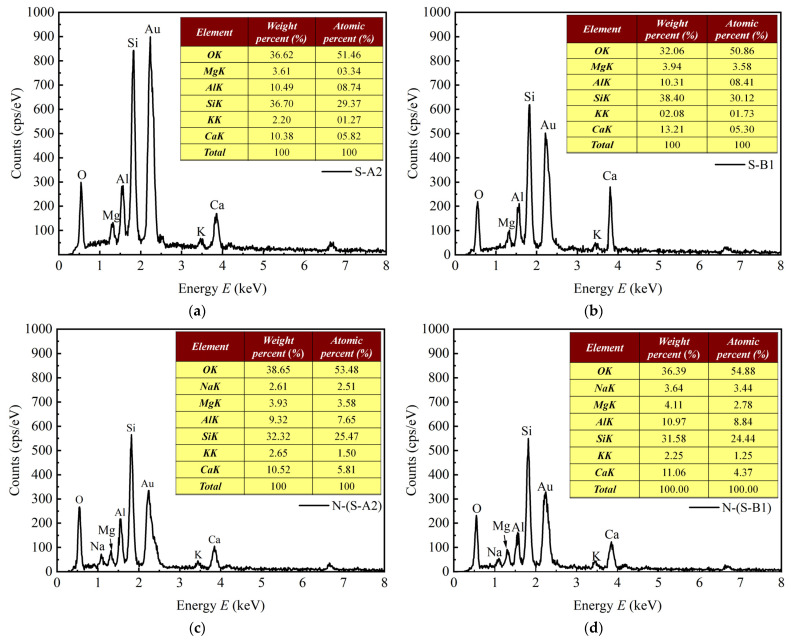
EDS patterns of (**a**) S-A2 and (**b**) S-B1 after 91 days of curing as well as (**c**) N-(S-A2) and (**d**) N-(S-B1) soaked in sodium sulfate solution for 28 days.

In Figure 15a–d, samples S-A2, S-B1, N-(S-A2), and N-(S-B1) were selected, and the energy dispersive spectrometer (EDS) spectra of the point sampling areas were plotted. The locations of the EDS sampling points are indicated within the rhomboid area in Figure 16a–d. The spectra and inserted tables present the chemical composition of the elements detected in the rhomboid area. The detected chemical components and their corresponding percentages are detailed in Table 8.

As illustrated in Figure 16a and Figure 16b, the weight percentage of Ca in samples S-A2 and S-B1 was 10.38 % and 13.21 %, respectively. The increase in weight percentage of Ca content from 10.38% to 13.21% was attributed to the partial replacement of GBFS by BG. This phenomenon was attributed to the presence of Al_2_O_3_ in the GBFS, while BG was predominantly rich in Ca. Figure 16c and Figure 16d demonstrated that solidified clay soaked in sodium sulfate solution exhibited different results, with weight percentages of Ca at 10.52 % and 11.06 %, respectively. Compared to unsoaked solidified clay, the weight percentage of Ca has decreased. This reduction may be attributed to the erosion of gel substances in solidified clay by the sodium sulfate solution, resulting in a change in the state of these gel substances [72]. The content of Ca in S-A2 and N-(S-A2) was 10.38% and 10.52%, respectively. The Ca/Si ratio increased from 0.283 to 0.325, while the Ca/(Si+Al) ratio was also enhanced from 0.22 to 0.253. This was attributed to the ionic exchange of SO_4_^2−^ in the sodium sulfate solution with the Ca^2+^ in C-S-H gel, which led to the further formation of AFt. The Ca/Si ratios of S-B1 and N-(S-B1) were similar numerically, with N-(S-B1) exhibiting a slightly higher value. The Ca/Si and Ca/(Si+Al) of S-B1 were 0.344 and 0.271, respectively, while those of N-(S-B1) were 0.35 and 0.266, respectively. From the change in values, the effect of the sodium sulfate solution on S-B1 was relatively small. It can be seen that the sodium sulfate solution affects the gel substance in S-B1 to a relatively small extent.

### 4.4. FTIR of Solidified Clay

**Figure 17:**
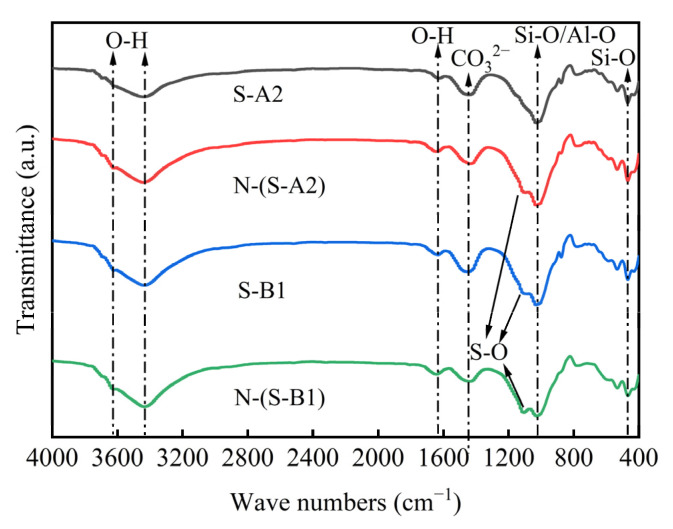
FTIR patterns of solidified clay.

To clarify the types of substances in solidified clay, FTIR was used to detect the functional groups and chemical bonds in solidified clay. Figure 17 shows the FTIR spectrum of solidified clay after 91 days of curing and 28 days of soaking in sodium sulfate solution. The spectra of the S-A2, S-B1, N-(S-A2), and N-(S-B1) indicated a peak at 860–1175 cm^−1^, which confirmed the presence of Si-O and Al-O bonds, further substantiating the existence of silicate and aluminate anion groups [73]. In addition to the stretching vibrations of Si-O, the bands of S-A2, S-B1, N-(S-A2), and N-(S-B1) at 870 cm^−1^ were attributed to the bending modes of CO_3_^2−^, which were typically reported around 875 cm^−1^ [74]. S-A2, S-B1, N-(S-A2) and N-(S-B1) exhibited stretching vibrations in the range of 1320–1530 cm^−1^, which were characteristic of the asymmetric stretching vibration of CO_3_^2-^, indicating the presence of CO_3_^2-^ in solidified clay. The stretching vibrations of the CO_3_^2−^ further confirmed the presence of calcite. Under the influence of alkaline substances such as MgO, the Si-O and Al-O bonds in the matrix break and reassemble, thereby promoting the formation of hydration products, including C-S-H gel and AFt crystals. The figure indicated that the produced phases exhibited their main peaks between 980 and 1020 cm^−1^, which were attributed to the presence of hydration products, such as C-S-H [75]. Characteristic peaks were observed near 1647 cm^−1^, while broad peaks appeared in the range of 3300–3800 cm^−1^. These peaks correspond to the stretching vibrations of the O-H group, indicating the presence of OH^−^ anions [76]. The characteristic peak near 473 cm^−1^ mainly corresponded to the asymmetric bending vibration of the Si-O-Si bond in silicate materials. In the spectral curves of S-B1, N-(S-A2), and N-(S-B1), several less pronounced smaller peaks were observed. Analysis of the peak positions revealed a correlation between these peaks and the presence of S-O. Based on the characteristics of the needle-like hydration products observed with a scanning electron microscope (Figure 15), it could be inferred that these products were CaSO_4_·2H_2_O or AFt crystals.

### 4.5. TGA of Solidified Clay

**Figure 18:**
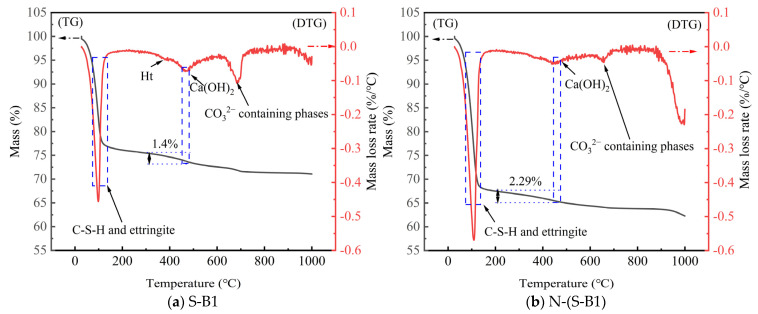
TGA test result of solidified clay with different conditions.

Through UCS and STS tests, S-B1 has a higher strength. To further clarify the hydration products of S-B1 and N-(S-B1), TGA tests were conducted, and the thermogravimetric (TG) and derivative thermogravimetric (DTG) results are shown in Figure 18.

The mass loss observed from room temperature to 100 °C primarily corresponded to the free water in the sample, while the mass loss occurred between 100 and 1000 °C was predominantly attributed to the hydration products. The TG curve indicated that the overall mass of S-B1 and N-(S-B1) decreased by 28.93% and 37.73%, respectively, during the temperature range of 30–1000 °C. By combining the TG curve and the DTG curve, it could be concluded that the mass loss observed in the temperature range of 50–110 °C primarily resulted from the loss of crystallization water in C-S-H gel and AFt crystals, as well as the evaporation of natural moisture [77]. The mass loss observed in the temperature range of 450–480 °C was attributed to the loss of crystal water in Ca(OH)_2_ [78,79]. From the DTG curve, it could be observed that the Ht gel exhibited a heat absorption peak at 400 °C. In the temperature range of 450–480 °C, the mass loss of S-B1 was 1.4%, while the mass loss of N-(S-B1) was 2.29%. This indicated that exogenous SO_4_^2−^ reacted with solidified clay through hydration to produce AFt crystals, while the crystals of Ca(OH)_2_ were released, which was consistent with the observations presented in Equation (6). The sodium sulfate solution has affected the gel substances of solidified clay, leading to a transformation in the types of gel substances, thereby affecting the mechanical strength of solidified clay. The DTG curve indicated that at a temperature of 600 °C, carbonate (CO_3_^2−^) exhibited an endothermic peak. This suggested that CO_3_^2-^ may have formed when solidified clay was exposed to CO_2_ in the air.

## 5. Conclusions

First, the effect of the binder materials on the performance of solidified clay was evaluated by mechanical tests. Second, the durability of solidified clay was evaluated using wet–dry cycles and soaking in sodium sulfate solution. Finally, the hydration products of solidified clay and their types were identified through microscopic analysis. Based on the experimental results and analysis, the following conclusions can be drawn:

(1) At curing times of 7 days, 28 days, and 91 days, the UCS of S-B1 increased by 49.27%, 41.02%, and 36.55% compared to S-A2, respectively. Under the same curing time, the STS of S-B1 increased by 78.65%, 24.88%, and 12.25% compared to S-A2. Since B1 contained GBFS, MgO, and BG, whereas A2 comprised only GBFS and MgO, the components in B1 were able to interact synergistically. In comparison to A2, the quantity of gel substances generated by hydration was greater in B1, resulting in the strength of S-B1 exceeding that of S-A2.

(2) The residual strength coefficients of solidified clay during wet–dry cycles were closely associated with the type of binder materials (A2 and B1), the curing times (7, 28, and 91 days), and the dosage of the binder materials (10% and 15% of the mass of the dry clay, respectively). The residual strength coefficients of S-B1 consistently exceeded those of S-A2. The UCS of solidified clay decreased with an increasing number of wet–dry cycles, with the reduction in UCS from 0 to 6 cycles being significantly greater than that from 6 to 10 cycles.

(3) The UCS of S-A2 and S-B1 decreased by 49.1% and 29.8%, respectively, compared to their values before soaking. The UCS of S-B1 decreased to a lesser extent compared to S-A2, primarily because the components in B1 were able to hydrate and form AFt crystals prior to soaking in sodium sulfate solution. AFt crystals usually maintain stability in sulfate solutions, which helps improve the sulfate resistance of solidified clay. Therefore, the incorporation of BG supplies the raw material necessary for the formation of AFt, resulting in S-B1 demonstrating superior resistance to sulfate attack compared to S-A2.

(4) The hydration products of S-A2 and S-B1 primarily comprised needle-like C-S-H gel and flaky Ht gel, whereas the hydration products of S-B1 also included AFt crystals. The gel substance of S-B1 was distributed between the particles in the form of agglomerates after soaking in sodium sulfate solution. The interlocking growth of C-S-H gel and AFt crystals provides effective structural support. BG promoted the formation of AFt crystals, while MgO facilitated the generation of C-S-H gel.

## Figures and Tables

**Table 1:** Physical properties of SC.

Material	Physical Property						
	Natural Moisture Content (%)	Specific Gravity	Total Density (g/cm^3^)	Liquid Limit (%)	Plastic Limit (%)	Plastic Index (%)	USCS Classification
SC	70	2.68	1.68	42.5	22.6	19.9	Lean clay (CL)

Note: USCS = unified soil classification system [36].

**Table 2:** Physical properties of OPC.

Fineness (%)	Setting Time (min)		Loss on Ignition (%)	Normal Consistency (%)	Density (g/cm^3^)	Specific Surface Area (m^2^/kg)	Compressive Strength (MPa)	
	Initial	Final					3 d	28 d
3.1	192	372	4.5	28	3.07	351	24.1	48.8

**Table 3:** Physical properties of GBFS, MgO, and BG.

Material	Particle Size Range Content (%)			Specific Surface Area (cm^2^/g)	Apparent Specific Gravity (g/cm^3^)	pH Value
	<2 μm	2 μm~75 μm	>75 μm			
GBFS	15.56	83.26	1.18	4998	2.87	11.12
MgO	19.87	79.91	0.22	2795.12	3.12	10.56
BG	6.98	63.18	29.84	2498.15	2.73	6.8

**Table 4:** Major chemical compositions (wt.%) of SC, GBFS, MgO, BG, and OPC.

Materials	Composition Content											
	SiO_2_	Al_2_O_3_	CaO	Fe_2_O_3_	SO_3_	MgO	Na_2_O	K_2_O	P_2_O_5_	TiO_2_	Mn_2_O_3_	Others
SC	72.1	17.5	0.43	4.2	2.9	0.9	0.54	1.4	/	0.01	0.02	/
GBFS	35	14.1	40.1	5.29	2.51	1.1	0.8	0.7	/	0.06	0.19	0.15
MgO	/	1.12	0.55	0.12	0.11	95.3	0.1	0.01	0.22	0.12	0.15	2.2
BG	2.09	0.89	35.1	0.65	52.87	0.67	/	0.12	0.19	0.17	0.1	7.15
OPC	25.6	13.1	45	3.55	3.55	0.85	0.63	0.25	0.16	0.26	0.13	6.92

**Table 5:** Design of binder materials.

Solidified Clay	Binder Materials	Percentage of GBFS, MgO, and BG in the Binder Materials (%)		
		GBFS	MgO	BG
S-A1	A1	90	10	-
S-A2	A2	80	20	-
S-A3	A3	70	30	-
S-A4	A4	60	40	-
S-A5	A5	50	50	-
S-B0	B0	80	20	-
S-B1	B1	60	20	20
S-B2	B2	40	20	40
S-B3	B3	20	20	60

**Table 6:** Mechanical properties test scheme.

Test Project	Sample Size (mm)	Curing Time (d)	Number of Cycles (d)	Soaking Time (d)
UCS	39.1 × 80	7, 28, 91	/	/
STS	50 × 50	7, 28, 91	/	/
Dry and wet cycle	39.1 × 80	28, 91	1, 2, 3,4, 5, 6, 7, 8, 10	/
Sodium sulfate solution soaking	39.1 × 80	91	/	1, 3, 7, 14, 28

**Table 7:** Microscopic test scheme.

Test Project	Solidified Clay	Curing Time (d)	Soaking Time (d)
XRD	S-OPC, S-A1, S-A2, S-A5, S-B1, S-B2, S-B3	91	/
	N-(S-A2), N-(S-B1)	/	28
SEM, EDS, FTIR	S-A2, S-B1	91	/
	N-(S-A2), N-(S-B1)	/	28
TGA	S-B1	91	/
	N-(S-B1)	/	28

**Table 8:** Weight percentage and ratio of elements in solidified clay.

Samples	Ca (%)	Si (%)	Na (%)	Ca/Si	Ca/Al	Ca/(Si+Al)
S-A2	10.38	36.7	/	0.283	0.99	0.22
N-(S-A2)	10.52	32.32	2.61	0.325	1.128	0.253
S-B1	13.21	38.4	/	0.344	1.28	0.271
N-(S-B1)	11.06	31.58	3.64	0.35	1.1	0.266

## Data Availability

The original contributions presented in this study are included in the article. Further inquiries can be directed to the corresponding author(s).
